# Heart’s Dangerous Symphony: Torsade De Pointes Unleashed by Gitelman Syndrome-Induced Hypomagnesemia

**DOI:** 10.7759/cureus.44464

**Published:** 2023-08-31

**Authors:** Iyad Y Idries, Muhammad Azhar, Ruchi Yadav, Anna Nevolina, Abid Ullah, Avtar Sur, Iryna Zadoretska, Moshe Gunsburg

**Affiliations:** 1 Internal Medicine, Brookdale University Hospital Medical Center, New York, USA; 2 Nephrology, Brookdale University Hospital Medical Center, New York, USA; 3 Hematology and Oncology, Brookdale University Hospital Medical Center, New York, USA; 4 Hematology and Oncology, Institute of Blood Pathology and Transfusion Medicine of the National Academy of Medical Sciences of Ukraine, lviv, UKR; 5 Electrophysiology, Brookdale University Hospital Medical Center, New York, USA

**Keywords:** ventricular arrhythmias (vas), wet beri-beri syndrome, prolonged qtc interval, torsades de pointes (tdp), renal tubular disorder, cardiopulmonary resuscitation, fatal arrhythmia, gitelman syndrome, hypomagnesemia, cardiac arrest

## Abstract

Gitelman syndrome (GS) is a rare autosomal recessive salt-losing renal tubular disorder associated with a mutation of SLC12A3 or CLCNKB genes which encodes the thiazide-sensitive sodium-chloride co-transporter (NCCT) in the distal renal tubule. It is inherited as an autosomal recessive disorder. Hypokalemia, metabolic alkalosis, hypomagnesemia, hypocalciuria, and renin-angiotensin-aldosterone system (RAAS) activation are characteristics of GS. GS is often misdiagnosed or underdiagnosed owing to its low incidence and lack of awareness. Its prevalence is estimated to be around 1-10 per 40,000 people. We report a case of cardiac arrest secondary to torsade de pointes (TdP) because of GS-induced hypomagnesemia. Our case highlights the importance of clinicians being aware of the potential electrolyte abnormalities and complications associated with GS, as it can lead to catastrophic consequences if not identified and corrected earlier.

## Introduction

Presented is a noteworthy case illustrating the intricate interplay between Gitelman syndrome (GS), a salt-wasting tubulopathy, and torsade de pointes (TdP), a life-threatening arrhythmia [1]. The patient's recurrent syncope episodes were attributed to TdP, which arose from hypomagnesemia secondary to GS. This syndrome, characterized by electrolyte imbalances, was underscored by recurring hypomagnesemia during prior admissions, augmented urinary magnesium excretion, and reduced calcium levels during the present hospitalization. TdP, characterized by polymorphic ventricular tachycardia and prolonged QT interval, prompted various interventions, including synchronized cardioversion and magnesium supplementation. What makes this case unique is the intricate connection between GS's electrolyte perturbations and the resultant arrhythmia emphasizes the critical importance of early recognition and management of electrolyte disorders in preventing life-threatening complications.

## Case presentation

We describe a case of a 28-year-old female who presented to the emergency department initially with unwitnessed seizures. She had a past medical history of alcohol use disorder, cocaine abuse, and a history of five previous hospitalizations for syncope attributed to unwitnessed seizures. During those hospitalizations, labs were significant for hypomagnesemia as low as 0.5 mg/dL. After retrospective reviews of previous admission EKGs, the patient was found to have QTc prolongation, which was attributed to alcohol consumption.

On arrival, her vital signs were stable, and her physical examination was remarkable for a scaling papula-macular hyperpigmented rash with scaling all over the chest, arms, and legs, which was later associated with atopic dermatitis since childhood. Her initial workup for syncope, including a complete blood count, comprehensive metabolic panel, and head CT scan, was remarkable for low magnesium of 0.5 mg/dL (normal range 1.7-2.2 mg/dL) and calcium of 4.7 mg /dL (normal range 8.5-10.5 mg/dl).

**Figure 1:**
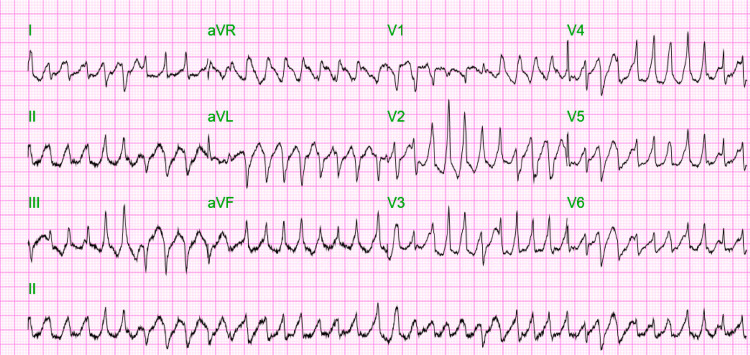
TdP in the setting of severe hypomagnesemia

**Figure 2:**
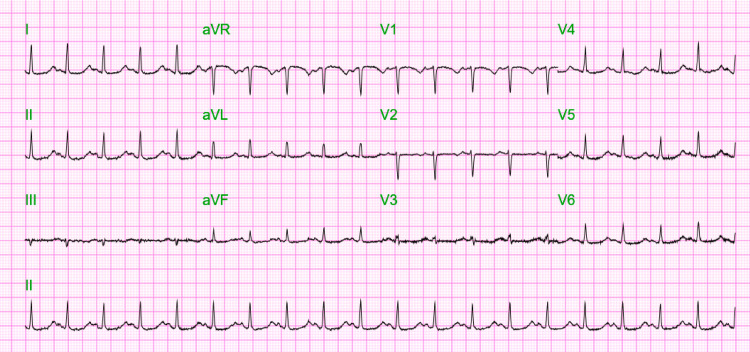
Severely prolonged Qtc in the setting of severe hypomagnesemia

An EKG revealed a heart rate of 230 beats/min and features consistent with TdP (Figure 1). Four grams of IV magnesium supplementation was administered, but despite this, the patient deteriorated. She became hypotensive to 70/40 mm Hg, and the EKG showed ventricular tachycardia. The patient was sedated with etomidate 2 mg/mL injection and received multiple synchronized cardioversion with a temporary response, receiving eight shocks (2 at 200 Joules and six dual sequential 200 Joules). Within that time, she developed multiple ventricular dysrhythmias, including polymorphic ventricular tachycardia and an episode of ventricular fibrillation. However, she never became pulseless; systolic blood pressure remained at about 70 mm Hg. Esmolol was considered, but lidocaine was more readily available, and a 100 mg IV bonus of lidocaine was given with conversion to sinus rhythm. Repeat EKG revealed sinus rhythm with markedly prolonged QTc 619 ms (Figure 2). The patient was intubated and placed on mechanical ventilation for airway protection, given depressed mental status.

During the further course, persistent hypomagnesemia with a level of magnesium remaining below 2 mg/dl despite continuous IV supplementations of more than 10 grams of magnesium daily was observed. Further analysis showed that the patient also had multiple hypomagnesemia episodes during previous admissions to the hospital or outpatient visits, with magnesium levels in a range of 0.5 to 1.5 mg/dl. Further subsequent analysis of 24-hour urine chemistries revealed high excretion of magnesium (>120 mg/24 hours), 24-hour urine calcium significantly low (<17.3 mg, average value 100-300 mg/24 hr), and urine chloride 242 mg/24 hours (chloride 24 hours was collected after starting treatment with potassium-sparing diuretic). Elevated urine excretion of magnesium, hypomagnesemia, and hypocalciuria was suggestive of GS, and the patient was started on potassium-sparing diuretic spironolactone 50 mg daily but was switched then to amiloride 5 mg daily as it has less endocrinological side effects. As a result of starting treatment with a potassium-sparing diuretic, requirements for magnesium supplementation significantly decreased, and serum magnesium levels improved. The patient was then successfully extubated. Genetic analysis for SLC12A3 gene mutation was not performed to establish the diagnosis of GS as it would not affect the management. The patient denied any family history of sudden deaths, arrhythmias, or kidney problems. The team recommended that the patient notify her family members of the possible GS as they may also be at risk for lethal cardiac arrhythmias.

On examination, the patient had a scaling rash all over her body, suspicious of vitamin deficiency vs. childhood atopic dermatitis, which was later confirmed to be vitamin B1 (thiamine) and B6 (pyridoxine) deficiency 2.6 ug/L (normal range 3.4-65 ug/L). Persistently increased lactate levels subsided after treatment with thiamine, proving high lactate to be secondary to thiamine deficiency.

Echocardiography (ECHO) showed severely decreased left ventricular systolic function, ejection fraction of 25-30%, akinesis of the mid-segment of the lateral and posterior wall, and possible akinesis/severe hypokinesis of the basal segments of the inferior, posterior, and lateral walls. Repeat ECHO done after two weeks showed improvement of ejection fraction to normal, which indicates initial low ejection fraction was due to acute hemodynamic insult and tachycardia because of TdP and other possibilities that could be related to wet beriberi syndrome or Takotsubo syndrome.

Additionally, a CT scan of the abdomen showed signs of cholecystitis, and a hepatobiliary iminodiacetic acid scan revealed suspected biliary obstruction at the level of the cystic duct; thus, a percutaneous cholecystostomy tube was placed by interventional radiology. A CT scan of the abdomen also demonstrated an atrophic pancreas. Laboratory findings were significant for low fecal elastase, a sign of chronic pancreatitis, and pancreatic enzyme supplementation was started.

## Discussion

GS is a rare, autosomal recessive, salt-losing renal tubular disorder associated with a mutation of the SLC12A3 gene, which encodes the Na-Cl co-transporter (NCCT) [1]. It is characterized by hypokalemia, metabolic alkalosis, hypomagnesemia, hypocalciuria, and renin-angiotensin-aldosterone system (RAAS) activation [2]. Different SLC12A3 variants may lead to phenotypic variability and severity, presenting as a spectrum of varied clinical presentations of GS [3]. Its prevalence is estimated at around 1-10/40,000 people [4]. GS is often missed due to low incidence and lack of awareness of medical providers. Patients with GS have clinical manifestations, including salt cravings, muscle weakness, cramps, fatigue, dizziness, nocturia, and thirst [5,6]. GS has been described with a spectrum ranging from muscle weakness to severe manifestations, including heart failure and ventricular arrhythmia [7]. Eliminating other causes of hypomagnesemia and hypocalcemia, such as metabolic alkalosis, chronic alcohol use, persistent vomiting, and extensive diuretic use, is essential.

In many reports, the diagnosis of GS was established on clinical rather than genetic grounds, potentially creating confusion with other related disorders [8]. However, diagnosis can be suspected with the following laboratory findings: hypokalemia and hypomagnesemia due to persistent renal losses, metabolic alkalosis, elevated urinary chloride excretion, and decreased calcium excretion [9]. Reduced sodium chloride reabsorption, blood volume, and renal salinization lower the blood pressure, activating the RAAS system and increasing aldosterone and renin levels, leading to hypokalemia and metabolic alkalosis. Decreased urinary calcium in GS patients may be associated with abnormal Na+/Cl- combined transport, weakening the intracellular Cl- super activation, increasing Ca2+ reabsorption, and decreasing urinary calcium [10]. The presented case highlights the complexity of hypomagnesemia etiology as multiple medical conditions coexisted.

The laboratory values in this patient satisfied the criteria for the diagnosis of GS, such as elevated excretion of magnesium and low excretion of calcium. Urine chloride was normal; however, it was obtained only after starting a patient on a potassium channel blocker. Twenty-four-hour urine sodium was also not obtained.

Hypomagnesemia in this patient was initially thought to be related to chronic alcohol use and malnourishment. However, extensive dose supplementation with intravenous magnesium was ineffective for a prolonged period, indicating another underlying etiology. Severely reduced ejection fraction could also incline to severe ventricular arrhythmias. However, in this case, the patient had TdP and QTc prolongation, strongly associated with hypomagnesemia. Besides, the ejection fraction returned to normal after treatment, which could be explained by wet beriberi in the setting of vitamin B1 deficiency or Takotsubo syndrome in the background of acute cardiac distress.

Hypokalemia and hypomagnesemia can significantly prolong the QTc interval and increase the susceptibility of the heart toward dangerous ventricular arrhythmias. It has been demonstrated that there is a tendency for prolonged QT intervals in patients with GS, with one study showing a prevalence as high as 40% [11].

The patient was previously diagnosed with type I bipolar disease and was supposed to be taking antipsychotic medications, which could also be related to prolonged QT interval. However, the patient stated being non-compliant and denied any outpatient medications for that condition. In one study, it was found that the most common clinical findings of hypomagnesemia were personality changes and depression; thus, the differentiation from psychiatric disease is essential [12]. In addition, it is crucial to remember that patients with QTc prolongation with a history of ventricular arrhythmias or recurrent syncope can be candidates for implantable cardioverter-defibrillator [13].

Newly diagnosed GS with severe electrolyte derangements was likely the cause of QTc prolongation and TdP arrhythmia in this patient. It is imperative to establish underlying disorders that may cause multiple clinical and laboratory abnormalities, as it will affect the prognosis and prevent fatal consequences.

## Conclusions

This case report highlights the rare but potentially life-threatening consequences of GS-induced hypomagnesemia. Though very uncommon GS can lead to severe electrolyte arrangement, which can be complicated with severe cardiac arrhythmias including but not limited to TdP.

The presented case also demonstrates the complexity of hypomagnesemia etiology, as multiple medical conditions coexisted, including chronic pancreatitis, vitamin B1 and B6 deficiency, and other electrolyte derangements. These overlapping conditions underscore the significance of a comprehensive evaluation in patients with multi-system involvement and diverse clinical presentations.

Recognizing the clinical manifestations of GS, particularly when associated with suspicious electrolyte imbalances like hypomagnesemia and hypokalemia, is crucial for prompt diagnosis and appropriate management. In this case, early identification and treatment of GS led to a significant improvement in the patient's cardiac function and overall outcome; careful analysis of the previous medical history is critical in increasing the clinical suspicion of this condition. This patient had five unwitnessed syncopes with negative EEG, which is more likely to be attributed to cardiac etiology. Clinicians should remain vigilant about the potential electrolyte abnormalities and complications associated with GS to prevent catastrophic consequences and optimize patient care. Moreover, further research and awareness among medical professionals are warranted to enhance the recognition and management of this rare genetic disorder.
